# Long-term Protection Against Herpes Zoster by the Adjuvanted Recombinant Zoster Vaccine: Interim Efficacy, Immunogenicity, and Safety Results up to 10 Years After Initial Vaccination

**DOI:** 10.1093/ofid/ofac485

**Published:** 2022-10-23

**Authors:** Ana Strezova, Javier Diez-Domingo, Kamal Al Shawafi, Juan Carlos Tinoco, Meng Shi, Paola Pirrotta, Agnes Mwakingwe-Omari, Zoster-049 Study Group, Michael Adams, Anitta Ahonen, Charles Andrews, Eugene Athan, Jose-Fernando BarbaGómez, Piero Barbanti, Elisabeth Barberan, Alain Baty, Niklas Bengtsson, Juergen Berger-Roscher, Katarina Berndtsson Blom, Jean Beytout, Loïc Boucher, Céline Boutry, Alain Boye, François Brault, Laurie Breger, Carles Brotons Cuixart, Covadonga Caso, Christine Cerna, Huey-Shinn Cheng, Hee Jin Cheong, Eun Ju Choo, Clóvis Cunha, Anthony L Cunningham, Dan Curiac, Benoit Daguzan, Antje Dahmen, Susan Datta, Maria Giuseppina Desole, Emmanuel Di Paolo, Marc Dionne, Petr Dite, Jan Dutz, John Earl, Tamara Eckermann, William Ellison, Jurij Eremenko, Meral Esen, Takashi Eto, Xavier Farrés Fabré, Cecil Farrington, Murdo Ferguson, Pierre André Ferrand, Matthew Finneran, David Francyk, Marshall Freedman, George Freeman, Antônio Tarcísio Freire, Peter Gal, Jean-Sebastien Gauthier, Beatrice Gerlach, Wayne Ghesquiere, Iris Gorfinkel, Christine Grigat, Josef Grosskopf, Monika Hamann, Pascal Hanrion, Paul Hartley, Andrew Hastie, Ken Heaton, Agnes Himpel-Boenninghoff, Thomas Horacek, David Shu Cheong Hui, Yieng Huong, Shinn-Jang Hwang, Giancarlo Icardi, Gabriele Illies, Junya Irimajiri, Wilson Jacob, Alen Jambrecina, Thiago Junqueira Avelino-Silva, George Kalema, Hyo Youl Kim, Christiane Klein, Uwe Kleinecke, Hans-Joachim Koenig, Satu Kokko, Pavel Kosina, Susanna Koski, Pekka Koskinen, Maximilian Kropp, Rie Kuroki, Outi Laajalahti, Pierre Lachance, Jacob Lee, Jin-Soo Lee, Peter Levins, Robert Lipetz, Bo Liu, Chiu-Shong Liu, Martin Lundvall, Luci Magimaiseelan, Mary Beth Manning, Jukka Markkula, Frederick Martin, Pyrene Martínez Piera, Damien McNally, Shelly McNeil, Guglielmo Migliorino, Beate Moeckesch, Stephan Morscher, Michael Mueller, Abul Kashem Munir, Cláudia Murta de Oliveira, Kenjiro Nakamura, Silvia Narejos Pérez, Yuji Naritomi, Patrice Nault, José Luiz Neto, Concepción Núñez López, Hiroaki Ogata, Åke Olsson, Pauliina Paavola, Dae Won Park, Janice Patrick, Karlis Pauksens, Mercè Pérez Vera, Lauri Peltonen, Georg Plassmann, Airi Poder, Terry Poling, Carol Pretswell, Samir Purnell-Mullick, George Raad, Michael Redmond, Philippe Remaud, Ernie Riffer, Patrick Robert, Alex Rodríguez Badia, Maria Luisa Rodríguez de la Pinta, Lars Rombo, Robert Rosen, Shari Rozen, Dominique Saillard, Bruno Salaun, Johan Sanmartin Berglund, Joachim Sauter, Axel Schaefer, Isabelle Schenkenberger, Juergen Schmidt, Bernhard Schmitt, Christian Schubert, Anne Schuind, Tino Schwarz, Ilkka Seppa, Edmund Kwok Yiu Sha, Gerald Shockey, Sylvia Shoffner, Elina Sirnela-Rif, Tommaso Staniscia, Hirohiko Sueki, Shin Suzuki, Denis Taminau, Guy Tellier, Manuel Terns Riera, Azhar Toma, Nicole Toursarkissian, Mark Turner, Benita Ukkonen, Anna Vilella Morató, Juergen Wachter, Brian Webster, Karl Wilhelm, Jonathan Wilson, Wilfred Yeo, Chong-Jen Yu, Toufik Zahaf, Irina Zahharova, Cristiano Zerbini

**Affiliations:** GSK, Rixensart, Belgium; FISABIO Fundación para el Fomento Investigación Sanitaria y Biomédica de la Comunitat Valenciana, Valencia, Spain; Modis, Belgium c/o GSK, Wavre, Belgium; Hospital General de Durango, Durango, Mexico; GSK, Rockville, Maryland, USA; GSK, Wavre, Belgium; GSK, Rockville, Maryland, USA

**Keywords:** adjuvanted recombinant zoster vaccine, immune response persistence, long-term efficacy, long-term safety

## Abstract

Approximately 10 years after vaccination with the recombinant zoster vaccine (RZV), an interim analysis of this follow-up study of the ZOE-50/70 trials demonstrated that efficacy against herpes zoster remained high. Moreover, the safety profile remained clinically acceptable, suggesting that the clinical benefit of the RZV in ≥50-year-olds is sustained up to 10 years.

Reactivation of the varicella-zoster virus, known as herpes zoster (HZ) or shingles, occurs most frequently after the age of 50 years [1]. However, HZ and its debilitating complications, including postherpetic neuralgia, are preventable by vaccination [2–4]. The adjuvanted recombinant zoster vaccine (RZV, *Shingrix*, GSK) was first approved in 2017 for the prevention of HZ in ≥50-year-olds. RZV is currently licensed in >40 countries worldwide, including the United States and the European Union, where it is also approved for adults aged ≥18 years who are at increased risk of developing HZ. In the 2 pivotal prelicensure phase III randomized clinical trials (ZOE-50 and ZOE-70), RZV demonstrated 97% and 90% efficacy against HZ in adults aged ≥50 and ≥70 years over a median follow-up of 3.1 and 3.7 years, respectively [3, 4]. To evaluate the durability of the efficacy, immunogenicity, and safety of RZV, a long-term follow-up (LTFU) study (ZOE-LTFU) was initiated, which enrolled all eligible recipients of at least 1 dose of RZV from ZOE-50/70 who were willing to participate. ZOE-LTFU is planned to follow participants for 6 additional years, starting at ∼5 years after vaccination with RZV [5].

In an interim analysis conducted after at least 2 years of follow-up in ZOE-LTFU, the efficacy of RZV against HZ was 84.0% for the period ranging from ∼5–7 years postvaccination. When evaluated from 1 month post–second RZV dose in ZOE-50/70 up to 8 years postvaccination, efficacy was 90.9%. In addition, humoral and cell-mediated immune responses plateaued at >6-fold above prevaccination levels, and the safety profile remained clinically acceptable [5].

Here we present the results of a second interim analysis based on data collected after at least 4 years of follow-up in ZOE-LTFU and up to 10 years postvaccination in ZOE-50/70.

## METHODS

### Study Design and Participants

In the ZOE-50/70 parent studies (NCT01165177, NCT01165229), adults aged ≥50 and ≥70 years were randomized 1:1 to receive 2 RZV or placebo doses 2 months apart [3, 4]. Participants receiving at least 1 RZV dose during ZOE-50/70 were eligible to participate in ZOE-LTFU (NCT02723773), and ∼50% enrolled [5].

ZOE-LTFU is an ongoing phase IIIB, open-label LTFU study of ZOE-50/70 conducted in 18 countries/regions (Australia, Brazil, Canada, Czech Republic, Estonia, Finland, France, Germany, Hong Kong, Italy, Japan, Republic of Korea, Mexico, Spain, Sweden, Taiwan, the United Kingdom, United States). The study started on April 16, 2016, and the data lock point (DLP) for this second interim analysis was on August 19, 2021, when participants had completed at least 4 additional years of follow-up and data accrual was complete through year (Y) 9. Results for Y10 are also included, although they are still incomplete at this DLP. Additional details, including a schematic representation of the study design, have been reported previously [5].

### Outcomes and Assessments

The primary objective of the study is to assess the efficacy of RZV against HZ over the total duration of ZOE-LTFU. Other main objectives include evaluation of efficacy of RZV against HZ from 1 month post–second RZV dose in ZOE-50/70 through the end of ZOE-LTFU (overall and by year postvaccination), persistence of humoral and cell-mediated immune (CMI) responses to RZV at each year postvaccination, and safety.

For efficacy analyses, HZ cases were ascertained rigorously as described previously [3, 4]. Humoral and CMI responses were determined in terms of anti–glycoprotein E (gE) antibody concentrations (expressed in milli-International Units per milliliter [mIU/mL]) and frequencies of gE-specific CD4[2+] T cells (CD4+ T cells expressing at least 2 of the 4 activation markers assessed: interferon-γ, interleukin-2, tumor necrosis factor–α, and CD40 ligand per 10^6^ CD4+ T cells) [6]. Long-term safety was evaluated in terms of serious adverse events related to study participation and HZ-related complications (eg, postherpetic neuralgia, disseminated HZ).

### Statistical Analyses

Efficacy in ZOE-LTFU was evaluated in the modified total vaccinated cohort (mTVC), consisting of participants who received both RZV doses and did not develop a confirmed HZ episode before 1 month post–dose 2 in ZOE-50/70. Participants with confirmed HZ in ZOE-50/70 were censored for the efficacy analysis. In ZOE-50/70 and ZOE-LTFU, humoral and CMI responses were assessed in participant subsets. Persistence of humoral/CMI responses in ZOE-LTFU was evaluated in the according-to-protocol cohort for persistence. Long-term safety was evaluated in participants enrolled for LTFU. Detailed criteria for inclusion in these cohorts have been presented previously [5].

Because more than half of the placebo recipients from ZOE-50/70 were also vaccinated with RZV in a subsequent study [7], historical control estimates of incidence rates from the ZOE-50/70 placebo groups were used to assess vaccine efficacy during ZOE-LTFU. Additional statistical considerations have been presented previously [5].

For the previous interim efficacy analysis [5], a yearly effect was used in the model to estimate the yearly incidence rate for the placebo group from ZOE-50/70 data. As this yearly effect does not apply to the ZOE-LTFU data, it was removed from the model used for the present interim analysis, and the yearly incidence rate in the placebo group was considered the overall rate in the placebo group of ZOE-50/70. This change in the model resulted in slightly different efficacy estimates for the ZOE-LTFU period in this compared with the previous interim analysis [5].

## RESULTS

### Study Participants

Of the 7413 participants enrolled for the long-term efficacy assessment, 7277 were included in the mTVC, and 813 and 108 were included in the according-to-protocol cohorts for humoral and CMI persistence. In the mTVC, the mean age at first vaccination in ZOE-50/70 was 67.3 (±9.4) years; 60.7% were women, and 76.5% of participants were of European ancestry. Demographic characteristics were comparable between the different cohorts [5].

### Long-term Efficacy

Over the ≥4-year follow-up in ZOE-LTFU, from a mean of 5.6 (±0.3) years to 9.6 (±0.3) years postvaccination, the interim analysis demonstrated 81.6% (95% confidence interval [CI], 75.2%–86.6%) efficacy of RZV against HZ (Table). When evaluated from 1 month post–dose 2 in ZOE-50/70 to a mean of 9.6 (±0.3) years postvaccination, the efficacy of RZV against HZ was 89.0% (95% CI, 85.6%–91.3%) in ZOE-LTFU participants. Annual vaccine efficacy estimates were ≥83.3% through Y8, 72.7% for Y9, and 73.2% for Y10 postvaccination.

**Table 1.** Vaccine Efficacy in the ZOE-50/70 Studies and ZOE-LTFU After at Least 4 Additional Years of Follow-up (mTVC)

	RZV				Historical Control^a^/Placebo Group in ZOE-50/70^b^				Vaccine Efficacy (95% CI), %	
	N	n	Sum of Follow-up Years	Incidence (per 1000 py)	N	n	Sum of Follow-up Years	Incidence (per 1000 py)		*P* value
Vaccine efficacy in ZOE-LTFU – primary objective (up to the data lock point for the second interim analysis in ZOE-LTFU)										
Overall^a^	7277	52	32 673.8	1.6	7277	283	32 673.8	8.7	81.6 (75.2–86.6)	*P* < .0001
Vaccine efficacy from 1 month post–dose 2 – secondary objective (up to the data lock point for the second interim analysis in ZOE-LTFU)										
Overall^a^	13 881	84	85 796.7	1.0	13 881	765	85 796.7	8.9	89.0 (85.6–91.3)	*P* < .0001
Year 1^b^	13 881	3	13 744.5	0.2	14 035	130	13 823.3	9.4	97.7 (93.1–99.5)	*P* < .0001
Year 2^b^	13 569	10	13 415.6	0.7	13 564	136	13 332.5	10.2	92.7 (86.2–96.6)	*P* < .0001
Year 3^b^	13 185	9	13 016.1	0.7	13 074	116	12 834.0	9.0	92.4 (85.0–96.6)	*P* < .0001
Year 4^b^	12 757	10	12 946.7	0.8	12 517	95	12 637.4	7.5	89.8 (80.3–95.2)	*P* < .0001
Gap between ZOE-50/70 and ZOE-LTFU										
Year 6^a^	7277	7	7210.2	1.0	7277	61	7210.2	8.5	88.5 (74.9–95.6)	*P* < .0001
Year 7^a^	7100	10	6995.8	1.4	7100	60	6995.8	8.6	83.3 (67.2–92.4)	*P* < .0001
Year 8^a^	6878	9	6762.9	1.3	6878	57	6762.9	8.4	84.2 (67.9–93.1)	*P* < .0001
Year 9^a^	6648	15	6487.6	2.3	6648	55	6487.6	8.5	72.7 (51.0–85.7)	*P* < .0001
Year 10^a,c^	6258	11	4869.1	2.3	6258	41	4869.1	8.4	73.2 (46.9–87.6)	*P* < .0001

Abbreviations: CI, confidence interval; mTVC, modified total vaccinated cohort; N, number of individuals included in each group; n, number of individuals with at least 1 confirmed herpes zoster episode; py, person-years; RZV, adjuvanted recombinant zoster vaccine; ZOE-LTFU, long-term follow-up study of ZOE-50/70.

a RZV vs matched historical controls from the placebo group in the ZOE-50/70 studies. The same N and follow-up period were considered for the historical control and vaccinated groups; n for historical controls represents the projected number of included placebo group participants from ZOE-50/70 with at least 1 confirmed herpes zoster episode based on the estimated incidence rate.

b RZV vs placebo recipients from the ZOE-50/70 trials. The follow-up ceased at the first occurrence of a confirmed herpes zoster episode, last contact date, or data lock point for this second interim analysis. All efficacy estimates are adjusted by region.

c At the data lock point for the second interim analysis in ZOE-LTFU, data collection for year 10 was still incomplete.

### Immunogenicity Persistence

**Figure 1.**
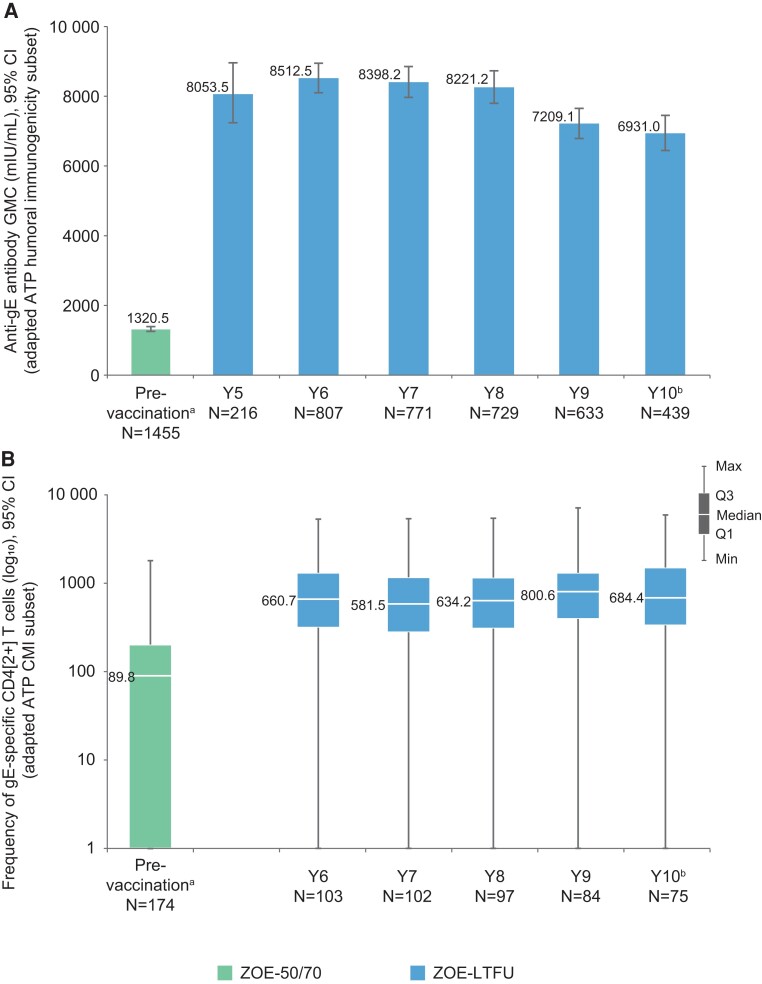
Persistence of humoral (*A*) and cell-mediated (*B*) immune responses to RZV up to the second interim analysis of ZOE-LTFU (ATP cohort for persistence). ^a^Prevaccination values from all RZV recipients in the humoral immunogenicity/CMI subsets in ZOE-50 and ZOE-70 [6]. Year 5 data for CD4[2+] T-cell frequencies are not shown because only 3 participants had available results for this analysis. ^b^At the data lock point for the second interim analysis in ZOE-LTFU, data collection for year 10 was still incomplete. Abbreviations: ATP, according-to-protocol; CI, confidence interval; CMI, cell-mediated immunity; gE, glycoprotein *E*; GMC, geometric mean concentration; CD4[2+] T cells, CD4+ T cells expressing at least 2 of 4 assessed activation markers (interferon-γ, interleukin-2, tumor necrosis factor–α, and CD40 ligand) per 10^6^ CD4+ T cells; Q1 and Q3, first and third quartiles, respectively; mIU/mL, milli-International Units per milliliter; N, number of participants with available results; RZV, adjuvanted recombinant zoster vaccine; ZOE-LTFU, long-term follow-up study of ZOE-50/70.

The prevaccination anti-gE antibody geometric mean concentration was 1320.5 (95% CI, 1253.6–1391.0) mIU/mL, and the postvaccination geometric mean concentrations remained >5-fold over this level across Y5–Y10 postvaccination (Figure 1*A*). The mean geometric increases of anti-gE antibody concentrations were ≥5.8 across this interval. The median prevaccination CD4[2+] T-cell frequency (interquartile range) was 89.8 (1.0–202.4) and plateaued at >6-fold over this level across Y5–Y10 postvaccination (Figure 1*B*).

### Long-term Safety

No deaths or other serious adverse events were considered causally related to vaccination by the investigator. Since the previous interim analysis [5], 3 participants reported HZ-related complications. Two participants aged 78 and 80 years at the time of diagnosis experienced postherpetic neuralgia at >9 years postvaccination. Pain was resolving when the first participant was lost to follow-up, and at the DLP for the present interim analysis for the second participant. The third HZ-related complication was disseminated HZ, occurring in an 81-year-old participant at ∼9 years postvaccination, and had resolved by the DLP for the present interim analysis.

## DISCUSSION

More than 95% of adults ≥20 years of age show serologic evidence of previous varicella-zoster virus infection, putting them at increasing risk for HZ and related complications throughout their lifetime [8]. Considering the increasing age-related risk, duration of protection in older adults is an important attribute; our LTFU study of >7000 adults with an average age of >67 years at vaccination showed that the efficacy against HZ remained high up to 10 years postvaccination.

The efficacy of RZV against HZ was 81.6% during the ≥4-year follow-up in ZOE-LTFU, ranging from a mean of 5.6 years up to 10 years postvaccination. When considering the period from vaccination in ZOE-50/70 up to 10 years, efficacy was 89.0%. Although vaccine efficacy plateaued from Y6 to Y8, it tended to decrease at Y9 and then remained stable through Y10.

Humoral immune responses during ZOE-LTFU plateaued through Y8, after which a decrease occurred through Y9, followed by a stabilization through Y10 at >5-fold over the prevaccination level. CMI responses remained stable at >6-fold above the prevaccination level in ZOE-LTFU through Y10. A similar trend in immune response kinetics has also been observed in adults ≥60 years of age [5, 9].

The strengths and limitations of our study have been detailed previously [5]. The limitations are related to the use of historical control group HZ incidence estimates for efficacy assessments, the fact that nearly half of ZOE-50/70 participants did not enroll in ZOE-LTFU, and the lack of data for Y5 efficacy estimations. Data accrual for Y10 is still ongoing, and precision of estimates for this time point will increase at the final analysis. The strengths include a long follow-up period (up to 10 years postvaccination at the DLP for this second interim analysis) and a more racially heterogeneous population for immunogenicity assessments compared with previous data [9].

## CONCLUSIONS

At ∼10 years after vaccination, the efficacy of RZV against HZ remained high, and immune responses to RZV remained >5-fold above prevaccination levels. In addition, the safety profile of RZV remained clinically acceptable. These data suggest that the clinical benefit of the RZV in adults aged ≥50 years is sustained up to 10 years after vaccination, which may reassure practitioners and consequently lead to increased vaccination coverage among those who are recommended to receive RZV.

## References

[1] Kawai K , Gebremeskel BG, Acosta CJ. Systematic review of incidence and complications of herpes zoster: towards a global perspective. BMJ Open 2014; 4:e004833. 10.1136/bmjopen-2014-004833 PMC4067812 24916088

[2] Oxman MN , Levin MJ, Johnson GR, et al A vaccine to prevent herpes zoster and postherpetic neuralgia in older adults. N Engl J Med 2005; 352:2271–84. 15930418 10.1056/NEJMoa051016

[3] Lal H , Cunningham AL, Godeaux O, et al Efficacy of an adjuvanted herpes zoster subunit vaccine in older adults. N Engl J Med 2015; 372:2087–96. 25916341 10.1056/NEJMoa1501184

[4] Cunningham AL , Lal H, Kovac M, et al Efficacy of the herpes zoster subunit vaccine in adults 70 years of age or older. N Engl J Med 2016; 375:1019–32. 27626517 10.1056/NEJMoa1603800

[5] Boutry C , Hastie A, Diez-Domingo J, et al The adjuvanted recombinant zoster vaccine confers long-term protection against herpes zoster: interim results of an extension study of the pivotal phase 3 clinical trials ZOE-50 and ZOE-70. Clin Infect Dis 2022; 74:1459–67. 34283213 10.1093/cid/ciab629 PMC9049256

[6] Cunningham AL , Heineman TC, Lal H, et al Immune responses to a recombinant glycoprotein E herpes zoster vaccine in adults aged 50 years or older. J Infect Dis 2018; 217:1750–60. 29529222 10.1093/infdis/jiy095 PMC5946839

[7] Ocran-Appiah J , Boutry C, Hervé C, et al Safety of the adjuvanted recombinant zoster vaccine in adults aged 50 years or older. A phase IIIB, non-randomized, multinational, open-label study in previous ZOE-50 and ZOE-70 placebo recipients. Vaccine 2021; 39:6–10. 33277059 10.1016/j.vaccine.2020.10.029

[8] Marin M , Guris D, Chaves SS, et al Prevention of varicella: recommendations of the Advisory Committee on Immunization Practices (ACIP). MMWR Recomm Rep 2007; 56:1–40. 17585291

[9] Hastie A , Catteau G, Enemuo A, et al Immunogenicity of the adjuvanted recombinant zoster vaccine: persistence and anamnestic response to additional doses administered 10 years after primary vaccination. J Infect Dis 2021; 224:2025–34. 32502272 10.1093/infdis/jiaa300 PMC8672743

